# Erdheim-Chester Disease Associated with Marginal Zone Lymphoma and Monoclonal Proteinemia

**DOI:** 10.1155/2011/941637

**Published:** 2011-10-20

**Authors:** Peter G. Pavlidakey, Alok Mohanty, Lisa J. Kohler, Howard J. Meyerson

**Affiliations:** ^1^Department of Pathology, University Hospitals Case Medical Center and Case Western Reserve University, 11100 Euclid Avenue, Cleveland, OH 44106-5056, USA; ^2^Office of the Summit Count Medical Examiner, 85 N. Summit St., Akron, OH 44308-1948, USA

## Abstract

Erdheim-Chester disease (ECD) is a rare non-Langerhans cell histiocytosis. We report a fatal case of ECD with extensive cardiac involvement associated with a marginal zone lymphoma and monoclonal proteinemia in a young man. This is the first reported association of ECD with a monoclonal gammopathy or a lymphoma.

## 1. Introduction

Erdheim-Chester disease (ECD) is a rare non-Langerhans cell histiocytosis with an unknown etiology similar to juvenile xanthogranulomatosis [1–8]. It is a multisystem disease most commonly involving bone, skin, orbit, pituitary, retroperitoneum, heart, and lung affecting middle-aged individuals with a slight male predominance [1–8]. Less commonly CNS, thyroid, testis, liver, and spleen are involved [2, 4, 5]. Characteristically the disease manifests as symmetric sclerotic bony lesions of the distal femur and proximal tibia and fibula [5]. Clinical outcome is determined by the extent and severity of extraskeletal disease, with most patients dying of disease secondary to pulmonary or cardiac involvement [1–8]. Coexisting neoplastic conditions have been described in rare occurrences. Mostly, they are associated with Langerhans cell histiocytosis. To date there have been no reports of monoclonal proteins or lymphoma accompanying ECD [9, 10]. Herein, we describe a young man with ECD associated with a marginal zone lymphoma and IgG lambda paraprotein.

## 2. Case Report

A 41 year old man was admitted to University Hospitals Case Medical Center for a two-week history of constitutional symptoms including arthralgias, night sweats, decreased appetite, dyspnea on exertion, and shortness of breath. Past medical history was significant for hypertension, thalassemia minor, hypogonadism, and recent history of uveitis. 

Laboratory findings at admission included the following: WBC 3.6 × 10^9^/L, RBC  5.52 × 10^12^/L, Hgb 11.1 g/dL Hct 33.1% MCV 60 fL, and Plt 193 × 10^9^/L. Chemistry tests performed one week before admission were normal except for a low albumin at 2.7 g/dL and calcium at 7.9 mg/dL. Notable abnormal laboratory findings during admission were elevated C-reactive protein at 4.0 mg/dL, sedimentation rate at 69, LDH 218 U/L, and Beta 2 microglobulin 8.0 mg/dL. Serum protein electrophoresis revealed a monoclonal IgG lambda paraprotein 0.8 g/dL. Angiotensin-converting enzyme was elevated at 156 U/L. Testing for viral infections including hepatic function tests, hepatitis B, HIV, parvovirus, and Epstein-Barr virus serology was negative. Serology for mycoplasma and syphilis was negative. Blood cultures failed to grow organisms. Studies for rheumatologic disorders were negative including an ANA panel, rheumatoid factor, and ANCA. 

 CT, MRI, and PET scans revealed extensive adenopathy and abnormal PET uptake within the lymph nodes of the neck, supraclavicular region, chest, abdomen and pelvis, and pericardial thickening suspicious for a lymphoproliferative disease. MRI of the orbits was unremarkable except for mild prominence of the optic disks. EKG and echocardiogram were unremarkable. 

A bone marrow biopsy was performed to investigate the cause of the paraproteinemia and revealed a hypercellular bone marrow (80%) with 5% mature-appearing plasma cells, normal trilineage hematopoiesis, mild erythroid hyperplasia, and microcytosis consistent with thalassemia minor (not shown). The plasma cells were lambda restricted by in situ hybridization staining. Flow cytometry performed on the marrow identified a small population of clonal plasmacytic cells that were surface light chain weakly lambda(+), CD19(+), HLA-DR(+), CD38bright, CD138(−), CD45(+) moderate to strong, CD20(−), CD5(−), and CD10(−) (not shown). The CD20(+) cells were not clonal. The phenotype was felt to be most consistent with a lymphoma with plasmacytic differentiation rather than a plasma cell dyscrasia [10]. 

**Figure 1:**
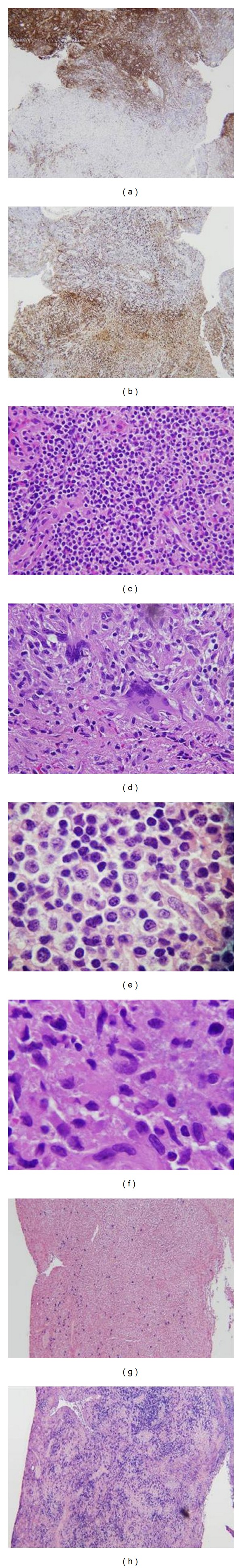
Morphologic features of lymph node. Images are from the antemortem lymph node biopsy. (a and b) CD20 and CD68 immunoperoxidase stains highlight the B-cell-rich and histiocyte-rich regions, the lymph node corresponding to areas involved by marginal zone B-cell lymphoma (a) and Erdheim-Chester disease (b), respectively, 100x magnification. (c and e) lymphoplasmacytic infiltrate present in the B-cell-rich areas, hematoxylin, and eosin, 200x and 400x magnification. (d and f) Histiocytic infiltrate with occasional Touton type giant cells in histiocyte-rich-regions, 200x and 1000x magnification. (g and h) In situ hybridization for kappa (g), and lambda (h), in lymphoplasmacytic areas of lymph node demonstrating lambda predominance, 100x magnification.

 Shortly thereafter, a cervical lymph node biopsy was performed that revealed an effaced lymph node with a dimorphic morphologic picture, Figure 1. A portion of the node demonstrated a diffuse proliferation of small lymphocytes with plasmacytoid features (Figures 1(a), 1(c), 1(e), 1(g), and 1(h)). The majority of cells in this region of the lymph node were CD20(+) with many lambda(+) plasmacytic cells. Flow cytometry revealed a small weakly lambda(+) CD19(+) cell population similar to that observed in the bone marrow (not shown). The findings were felt to be that of a lymphoma with plasmacytic differentiation, most likely a marginal zone lymphoma with extensive plasmacytic differentiation (P-MZL). 

Other regions of the lymph node demonstrated a diffuse proliferation of histiocytic cells with pale staining cytoplasm and bland nuclei (Figures 1(b), 1(d), and 1(f)). Only very rare giant cells were observed (Figure 1 (d)). Immunohistochemical staining revealed that the cells were CD68(+), CD1a(−), S100(−), CD31(+) weak, and lysozyme (+). By flow cytometry, an abnormal monocytic/histoicytic population was not identified. AFB and GMS were negative and culture of the lymph node did not grow bacteria or fungi. The exact significance of the histiocyte proliferation was unable to be determined from the biopsy.

The patient was subsequently started on prednisone with improvement of his symptoms and was discharged from the hospital with a follow-up appointment two weeks later. However, he expired suddenly at home three days after discharge. An autopsy was performed by the Summit County (Ohio) Medical Examiner.

**Figure 2:**
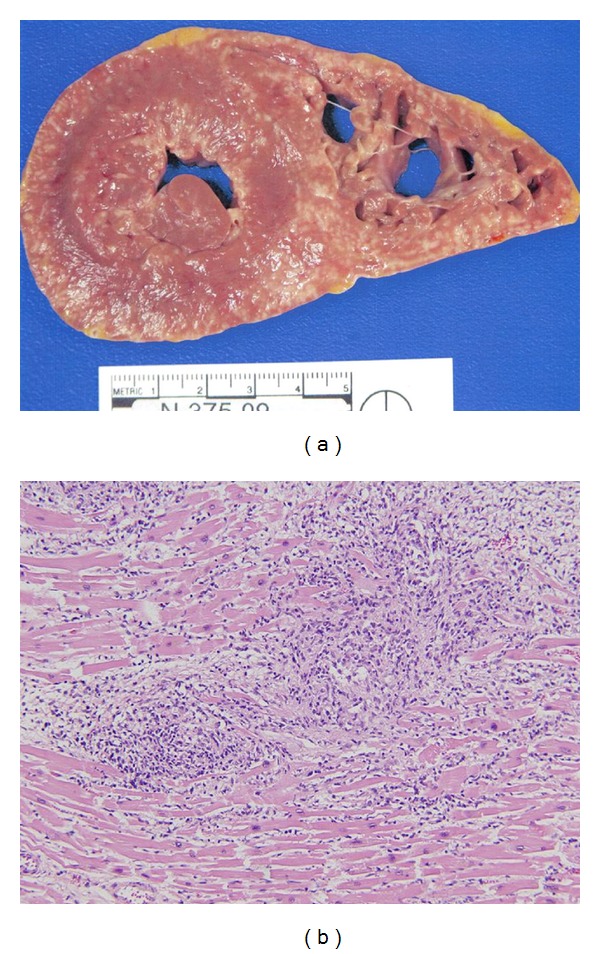
Gross and microscopic appearance of heart. Cross-section of heart, left, demonstrating extensive tan-yellow marbling of the myocardium due to infiltration by histiocytes. Microscopic appearance of heart, right. There was dense proliferation of histiocytes within the myocardium similar to that seen in the lymph node. Hematoxylin and eosin, 100x magnification.

 Autopsy findings demonstrated extensive myocardial and peripancreatic adipose tissue infiltration by histiocytic cells similar to that observed in the antemortem lymph node biopsy, Figure 2. The cardiac involvement was most impressive and also the cause of the patient's demise. The infiltrate was also seen focally in skeletal muscle, lymph nodes, and lung. Only rare multinucleated Touton-like giant cells were seen. There was no necrosis or caseation. The immunohistochemical profile of the histiocytic infiltrate in the postmortem sample was similar to that seen in the antemortem lymph node biopsy although S100 was focally, weakly positive (not shown). The pattern of infiltrate, the morphology, and the phenotype of the cells were diagnostic for Erdheim-Chester disease [1–8]. Lymphoma was not clearly evident in postmortem tissue sections.

## 3. Discussion

Erdheim-Chester disease (ECD) is a rare non-Langerhans cell histiocytosis with an unknown etiology similar to juvenile xanthogranulomatosis [1–8]. It generally affects middle-aged individuals with a slight male predominance [5]. Histologically, ECD is characterized by mononuclear infiltrate consisting of lipid laden or foamy histiocytes [1–8]. These histiocytes differ from Langerhans cells both immunohistochemically and microscopically as the monocytic infiltrate in ECD is positive for CD68, lacks reactivity to CD1a and the cells do not contain Birbeck granules [1–4, 6–9]. There is often a background of fibrosis with occasional Touton-type giant cells. It is unclear whether the histiocytes in the process are clonal. [1, 6–9]. The morphologic and phenotypic features of this patient's histiocytic proliferation, best illustrated in post-mortem material, were diagnostic for ECD. 

Prognosis of the ECD is dependent on visceral involvement with median survival roughly 3 years after diagnosis [5]. Many cases are not discovered until after death. Cardiac disease is not an uncommon cause of death in ECD. There are several cases of ECD involving the heart with gross and microscopic features similar to those presented here [3].

The differential diagnosis of ECD includes Langerhans cell histiocytosis, Rosai-Dorfman disease, histiocytic sarcoma, sarcoidosis, and granulomatous inflammation associated with infections. In this case, Langerhans cell histiocytosis was excluded by lack of staining for CD1a and Rosai-Dorfman disease, histiocytic sarcoma, and sarcoid by the morphology. Infectious causes were excluded based on cultures, serology, and histochemical stains. 

ECD was present concurrently with a clonal lymphoplasmacytic disorder in this patient. The neoplasm was best classified as marginal zone lymphoma with extensive plasmacytic differentiation (P-MZL). The clonal cells had an unusual CD19(+), CD20(−), CD38(bright +), CD45(+) moderate to bright, CD138(−), and weakly surface lambda(+) phenotype. The expression of CD19 and CD45 and lack of CD138 differs from that typically observed in plasma cell myeloma and is more in keeping with a plasmacytic lymphoma [10]. Additionally, lytic bone lesions were lacking. A lymphoplasmacytic lymphoma (LPL) was also considered although the nodal nature of the neoplasm and lack of a detectable mature B-cell antigen positive (CD20(+), CD22(+), and CD79b(+)) cell population in addition to the clonal plasmacytic cells favored a P-MZL [11, 12]. Paraproteins may be seen in up to 50% of MZLs, although less commonly than in LPL [13].

 An unusual feature of this patient was the coexistence of the B-cell lymphoma with ECD. ECD has only rarely been associated with other neoplasms almost always Langerhans cell histiocytosis [8, 9]. We could find no previous reports of a monoclonal gammopathy or lymphoma accompanying ECD. Interestingly, monoclonal proteinemia due to lymphoma or myeloma may insight a histiocytic proliferation termed crystal-storing histiocytosis [14]. This disorder is characterized by a proliferation of histiocytes with crystalline inclusions in their cytoplasm. The histiocytic proliferation may have a wide-spread tissue distribution. These cases are almost always due to kappa secreting plasmacytic disorders and are likely due to defective metabolism by histiocytes of the secreted paraproteins [14]. In the current case, intracellular crystalline deposits within the histiocytes were not seen, and this patient had a lambda light chain expressing paraprotein precluding the diagnosis of crystal-storing histiocytosis. Nonetheless, the coexistence of two unusual processes in this young man suggests a biologic relationship that may be akin to crystal-storing histiocytoses. Crystal-storing histiocytosis may accompany marginal zone lymphoma additionally supporting this hypothesis [15]. Moreover, clonality studies of histiocytes in ECD have given inconsistent results, and it remains unclear whether the cells in this disorder are neoplastic or reactive [1, 6–8]. Given the findings in this case, it may be of interest to evaluate additional patients with ECD for monoclonal gammopathies. 

In summary, we present a case of ECD and P-MZL accompanied by an IgG lambda monoclonal protein. This is the first report of this association. A paraprotein-induced histiocyte proliferation similar to that seen with crystal-storing histiocytosis is suggested as a unifying etiology. Evaluating patients with ECD for monoclonal proteins may be useful in determining whether a causal relationship exists between these disorders. 

## References

[1] Chetritt J, Paradis V, Dargere D (1999). Chester-Erdheim disease: a neoplastic disorder. *Human Pathology*.

[2] Sheu SY, Wenzel RR, Kersting C, Merten R, Otterbach F, Schmid KW (2004). Erdheim-Chester disease: case report with multisystemic manifestations including testes, thyroid, and lymph nodes, and a review of literature. *Journal of Clinical Pathology*.

[3] Haroche J, Amoura Z, Dion E (2004). Cardiovascular involvement, an overlooked feature of Erdheim-Chester disease: report of 6 new cases and a literature review. *Medicine*.

[4] Dickson BC, Pethe V, Chung CT (2008). Systemic Erdheim-Chester disease. *Virchows Archiv*.

[5] Veyssier-Belot C, Cacoub P, Caparros-Lefebvre D (1996). Erdheim-Chester disease: clinical and radiologic characteristics of 59 cases. *Medicine*.

[6] Gong L, He XL, Li YH (2009). Clonal status and clinicopathological feature of Erdheim-Chester disease. *Pathology Research and Practice*.

[7] Al-Quran S, Reith J, Bradley J, Rimsza L (2002). Erdheim-Chester disease: case report, PCR-based analysis of clonality, and review of literature. *Modern Pathology*.

[8] Tsai JW, Tsou JH, Hung LY, Wu HB, Chang KC (2010). Combined Erdheim-Chester disease and Langerhans cell histiocytosis of skin are both monoclonal: a rare case with human androgen-receptor gene analysis. *Journal of the American Academy of Dermatology*.

[9] Andrade VP, Nemer CC, Prezotti AN, Goulart WS (2004). Erdheim-Chester disease of the breast associated with Langerhans-cell histiocytosis of the hard palate. *Virchows Archiv*.

[10] Hussong JW, Perkins SL, Schnitzer B, Hargreaves H, Frizzera G (1999). Extramedullary plasmacytoma. A form of marginal zone cell lymphoma?. *The American Journal of Clinical Pathology*.

[11] Morice WG, Chen D, Kurtin PJ, Hanson CA, McPhail ED (2009). Novel immunophenotypic features of marrow lymphoplasmacytic lymphoma and correlation with Waldenström’s macroglobulinemia. *Modern Pathology*.

[12] Meyerson HJ, Bailey J, Miedler J, Olobatuyi F (2011). Marginal zone B cell lymphomas with extensive plasmacytic differentiation are neoplasms of precursor plasma cells. *Cytometry Part B—Clinical Cytometry*.

[13] Wöhrer S, Streubel B, Bartsch R, Chott A, Raderer M (2004). Monoclonal immunoglobulin production is a frequent event in patients with mucosa-associated lymphoid tissue lymphoma. *Clinical Cancer Research*.

[14] Lebeau A, Zeindl-Eberhart E, Müller EC (2002). Generalized crystal-storing histiocytosis associated with monoclonal gammopathy: molecular analysis of a disorder with rapid clinical course and review of the literature. *Blood*.

[15] Kusakabe T, Watanabe K, Mori T, Iida T, Suzuki T (2007). Crystal-storing histiocytosis associated with MALT lymphoma of the ocular adnexa: a case report with review of literature. *Virchows Archiv*.

